# Double osseous flaps for simultaneous midfacial and mandible reconstruction: Automation in surgical complexity within an entirely computerized workflow

**DOI:** 10.3389/fonc.2023.1103104

**Published:** 2023-02-03

**Authors:** Alessandro Tel, Daniele Bagatto, Salvatore Sembronio, Silvano Ferrari, Massimo Robiony

**Affiliations:** ^1^ Department of Maxillofacial Surgery, University Hospital of Udine, Udine, Italy; ^2^ Department of Neuroradiology, University Hospital of Udine, Udine, Italy; ^3^ Department of Maxillofacial Surgery, University Hospital of Parma, Parma, Italy

**Keywords:** virtual surgical planning, double bone flap, computerized automation, 3D printing, point-of-care, statistical shape modeling

## Abstract

**Introduction:**

Broad maxillofacial surgical resections involving both the midface and the mandible represent a challenge in terms of reconstruction. Although several papers have explored the possibility of simultaneously using two microsurgical flaps, reports on the implementation of a dual osseous flap strategy are limited, and mainly addressed to static anatomical reconstruction, regardless of functional implications. In particular, there is a lack in the literature of a unifying protocol which illustrates how technology including virtual planning, statistical shape modeling, virtual occlusion, 3D-printing and patient-specific implants can address the functional and accuracy needs required for an optimal reconstruction.

**Materials and methods:**

In this paper, the Authors present their preliminary experience in a two-center study, showing how broad maxillofacial defects, requiring a simultaneous reconstruction in both the mandible and the midface, can be successfully reconstructed using the combination of two osseous flaps in an automated sequence in which all steps are anticipately defined in a virtual plan, accounting for the optimal alignment of temporomandibular joint, predicting the final occlusion and defining a mandibular shape according to a statistical shape model.

**Results:**

Average RMSE for the iliac bone crest flap was of 3.2 ± 0.36 mm; for the fibula flap, RMSE value was of 2.3 ± 0.65 mm, for patient-specific implants, for mandibular prostheses the average RMSE was 2.46 mm with 0.76 mm standard deviation. Temporomandibular joint function increased when a TMJ prosthesis was placed.

**Conclusions:**

Double bone free flap is a valuable resource to reconstruct wide defects that simultaneously involve two thirds of the cranio-maxillo-facial skeleton, but a careful virtual planning study should be always performed before approaching this surgical option.

## 1 Introduction

In the last years, the improvement of microsurgery has led to significant advances in reconstructive surgery for skeletal defects. The customization of flap sculpting by virtually simulating precise correspondences between the defect size and the most appropriate flap conformation, and well as the possibility to bring such plans in the surgeon’s hands by 3D printing of specific surgical guides, has provided a further impulse to reconstructive microsurgery, fastening flap harvesting and positioning time, while improving outcomes. A variety of studies reported a dual flap strategy, where one osseous flap is generally used for the reconstruction of the bone framework, and another soft tissue flap is used to restore the skin or the mucosal lining, or simply to add volume after conspicuous resections (1). For the most complex defects, several publications report the use of virtual planning workflows to conform a single flap in convoluted spatial arrangements, allowing to optimize the donor flap to the recipient site, which is generally the midface due to its complex geometry including the maxillo-malar buttresses, the alveolar process and the orbit (2–4). However, for defects involving at the same time the midface and the inferior third, one single osseous flap might not be sufficient.

Currently, literature provides scant evidences on the simultaneous use of two osseous flaps: prolonged surgical time, risk of failure and technical complexity represent factors that have limited the adoption of a double bone-flap approach so far. In this respect, virtual surgical planning becomes an indispensable tool to anticipately define with maximum detail all the steps of surgery, from bone shaping, to insetting position and the choice of target vessels for anastomosis; likewise, 3D printing allows to construct surgical guides that assist the surgeon in the correct execution of each planning sequence, creating a fully automated workflow, which optimizes surgical time and increases accuracy and the functional outcomes.

The purpose of this study is to elucidate the technical aspects necessary to plan a simultaneous midfacial and mandible reconstruction with a double osseous free flap strategy, combining a fibula free flap with an iliac crest free flap. We describe an entirely automated sequence of virtual surgical planning and its application, emphasizing the importance of computerized simulation to achieve satisfactory accuracy and functional outcomes.

## 2 Materials and methods

This is a retrospective observational study performed in two centers with broad oncologic expertise, the University of Udine and the University of Parma. 3 patients were enrolled in this series between January 2021 and June 2022 and fulfilled the following requirements: surgically determined broad skeletal defect with simultaneous involvement of the midface and the mandible; simultaneous use of two osseous flaps for reconstruction; entirely digital workflow. All clinical and demographic features of patients are described in **Table 1**. We detail the entire workflow of anatomy digitalization, virtual surgical planning and operative sequences to illustrate how computer-guided steps can translate into automated workflows for optimal accuracy and functional restoration. Our institute’s local independent Institutional Review Board approved the study protocol (ID: IRB_45_2020).

**Table 1:** Demographic, clinical, and digital characteristics of patients enrolled in this study.

ID	Gender	Age	Pathology	Resection	Flap combination	Prosthesis	Components for computer-guided automation			Surgical complications	Accuracy (RMSE)		Follow up (TMJ opening)	
							VSP	guides	Occlusion and TMJ		flap	implant	3-month	6-month
1	Male	27	Fibrous dysplasia of the cranio-maxillo-facial skeleton	Subtotal mandibulectomy with right disarticulation and type 4 maxillary resection	Left fibula + right DCIA	SSM-shaped mandibular implant with right TMJ; 3-segment fibula flap, orbital PMMA implant	Virtual maxillectomy, vascular structures, mandible resection, flap sizing and inset, orbital reconstruction	Maxillary resection, mandible resection, DCIA harvest, fibula harvest, DCIA inset	Virtual wax-up; TMJ reconstruction with fossa and condyle prosthesis	None	3.1mm (DCIA) 2.2mm(fibula)	2.3mm (mandible implant) 2.1 (orbital implant)	28mm	35mm
2	Male	69	Clear-cell odontogenic carcinoma of the mandible with deformation of the midface	Subtotal mandibulectomy with TMJ preservation; type 3 maxillectomy	Right fibula + left DCIA	SSM-shaped mandibular implant, 2-segment fibula flap	Virtual maxillectomy, vascular structures, mandible resection, flap sizing and inset	Maxillary resection, mandible resection, DCIA harvest, fibula harvest, DCIA inset	Virtual wax-up	Intraoral dehiscence	3.6mm (DCIA) 1.7mm(fibula)	3.3mm (mandible implant)	24mm	32mm
3	Female	44	Chondroblastic osteosarcoma of the mandible with maxillary osteoradionecrosis	Segmental mandible resection with type 4 maxillectomy	Left fibula + right DCIA	Mirrored-shape mandibular implant, 1-segment fibula flap	Virtual maxillectomy, vascular structures, mandible resection, flap sizing and inset	Maxillary resection, mandible resection, DCIA harvest, fibula harvest, DCIA inset	Virtual wax-up	Wound dehiscence	2.9mm (DCIA) 3mm (fibula)	1.8mm (mandible implant)	30mm	28mm

### 2.1 Acquisition protocols

According to the concept of multilayer anatomical reconstruction (5), imaging protocols were directed to reproduce virtual anatomy both in the resection area and the two sites for bone flap harvest.

As for the resection area, the following imaging was acquired for the cranio-maxillo-facial region:

• multidetector CT scan with resolution of 768 x 768 voxel and slice thickness of 0.625mm was acquired and reconstructed in bone window density. These parameters were the most suitable to provide a trustful representation of bone anatomy, including thin areas with rarefaction due to pathological processes and partial volume effect.
• MR was acquired using a 3T machine. Contrast-enhanced T1-weighted, T2-weighted, volumetric interpolated breath-hold examination (VIBE), short-T1 inversion recovery (STIR) sequences were acquired as volumetric, with 512 x 512 voxel matrix and 1mm slice thickness. These acquisitions were crucial to characterize soft tissues and spatially define the pathological processes.
• 3D time-of-flight (TOF) sequence with spatial resolution 256x256 and 0.6mm slice thickness was acquired to reconstruct arterial vessels in the face and neck area
• MR venography was performed on a 3T machine to identify venous vasculature using a phased contrast sequence with 256 x 256 voxel matrix and 1mm slice thickness
• Patients furthermore underwent an intraoral scanning procedure to define dental cusps anatomy and use such data to reconstruct a virtual occlusion

As for the flap harvesting sites, for all anatomical regions the protocol was the same, including contrast-enhanced CT scan with 768 x 768 or at least 512 x 512 voxel matrix and a slice thickness of 0.625mm.

### 2.2 Digitalization of anatomy

**Figure 1:**
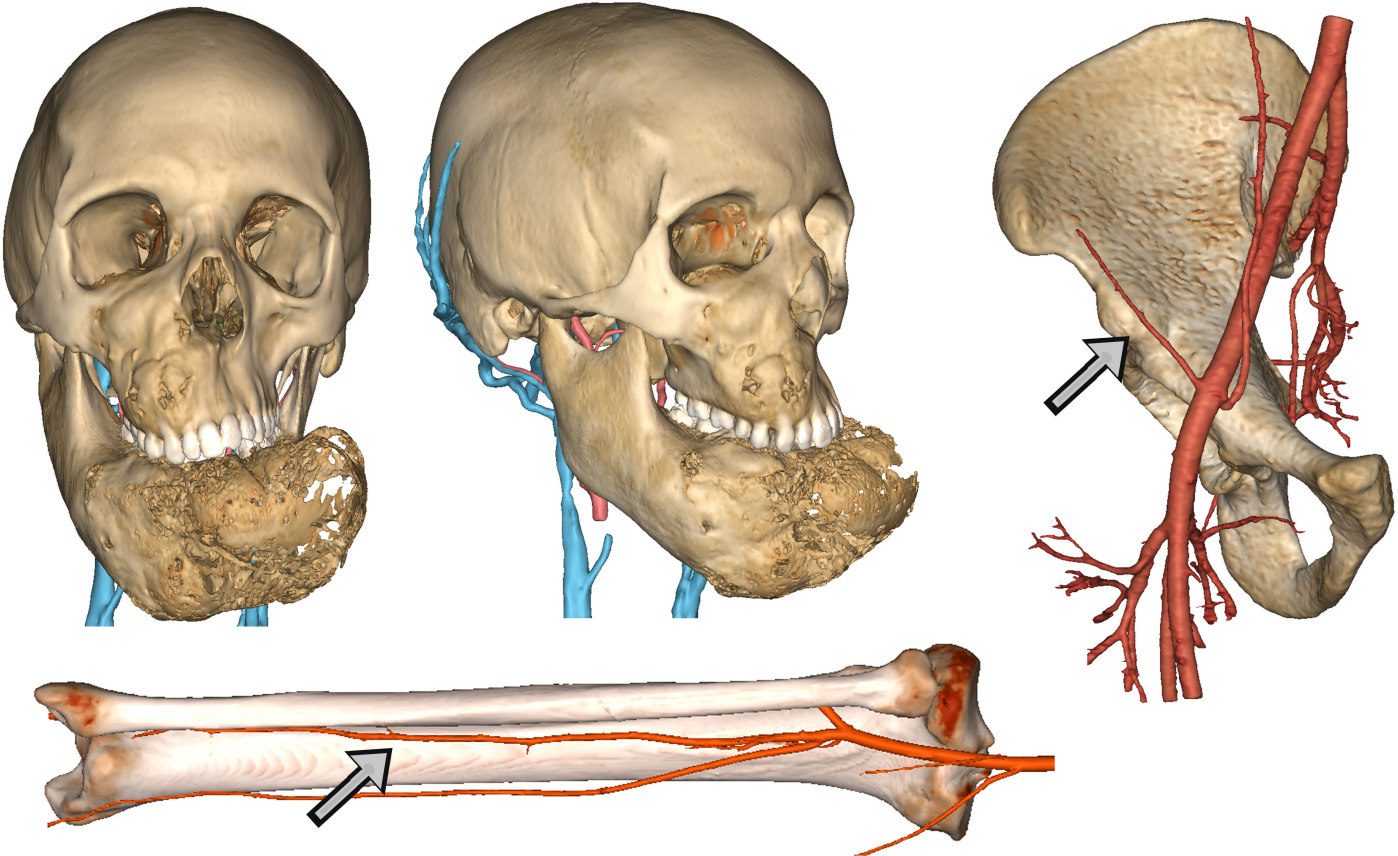
Detailed anatomical reconstruction of the craniomaxillofacial model of a fibrous dysplasia involving both the midface and the mandible with massive deformation. Associated, the reconstruction of arterial and venous vasculature is visible. For free flaps, virtual models of donor site were reconstructed with arterial vessels to evaluate the pedicle. Arrows show respectively the deep circumflex iliac artery and the fibular artery.

In the head and neck region, images were imported into Mimics v25 (Materialise, Leuven, BE) and coregistered within the same coordinate system. First, CT scan was segmented using a thresholding algorithm in the Hounsfield Unit (HU) range of bone, by applying a local selective improvement over thin structures, such as ethmoid and orbital walls. For tumors or pathological processes involving soft tissues, MR sequences were matched and semiautomatic methods based on AI (artificial intelligence)-powered smart brushes were used to accurately segment the tumor mass. A thresholding algorithm in the range of hyperintense values was applied to TOF sequences to yield an accurate representation of arterial vasculature; likewise, 3D phased-contrast venous MR were approached using detection methods based both on voxel contiguity and isointensity to reconstruct well defined 3D networks. Processing of segmentation masks was accurately accomplished especially around vessels to isolate relevant branches. As for flap donor sites, a baseline pre-contrast scan was used to reconstruct bone surfaces and was then coupled with a contrast-enhanced scan to reconstruct the vasculature and evaluate the position and length of the pedicle. **Figure 1** shows the result of anatomical 3D reconstruction.

### 2.3 Automation and sequencing: virtual planning translated into surgery

• Simulated resection

**Figure 2:**
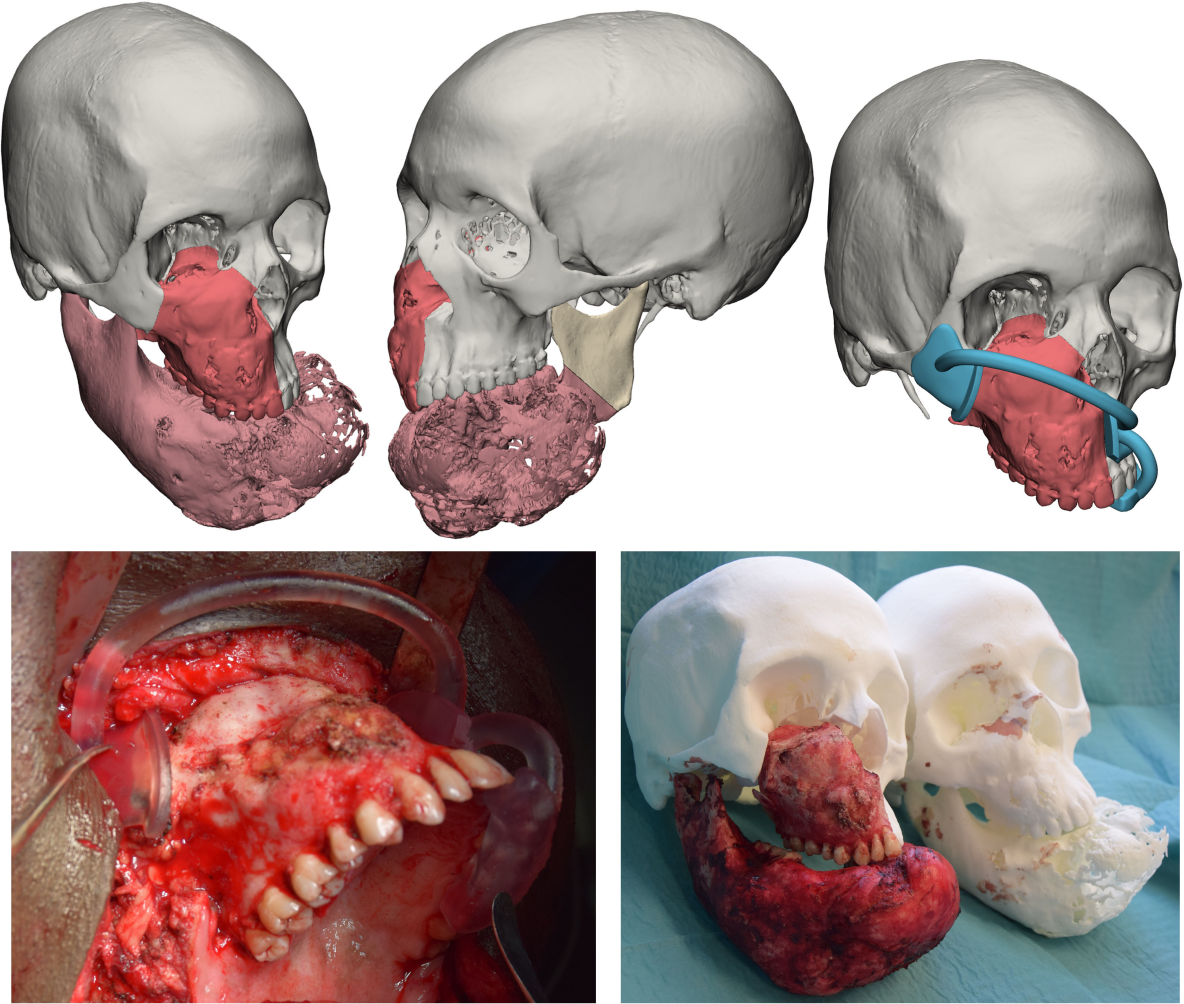
Top panel: simulated resection and design of a surgical guide. Bottom panel: intraoperative application of the surgical guide and assessment of resection accuracy by comparison with a 3D printed phantom.

Objects generated in Mimics were imported in 3-matic v 17 (Materialise, Leuven, BE). As all cases required a composite resection involving both the middle and the inferior third, a resection of the mandible and a maxillectomy were planned. The complex shape of maxillectomies was planned using a freehand brush poly-marking tool to draw complex shapes and define curved resection profiles without being limited to simple planes. A dual-fitting surgical guide was designed, allowing to simultaneously engage dental cusps using a dental splint appendix, and bone as well using patient-fitted surfaces (**Figure 2**). For subtotal mandibulectomies, the resection was easily defined by positioning a cutting plane.

• Definition of a statistical shape model for mandible reconstruction

**Figure 3:**
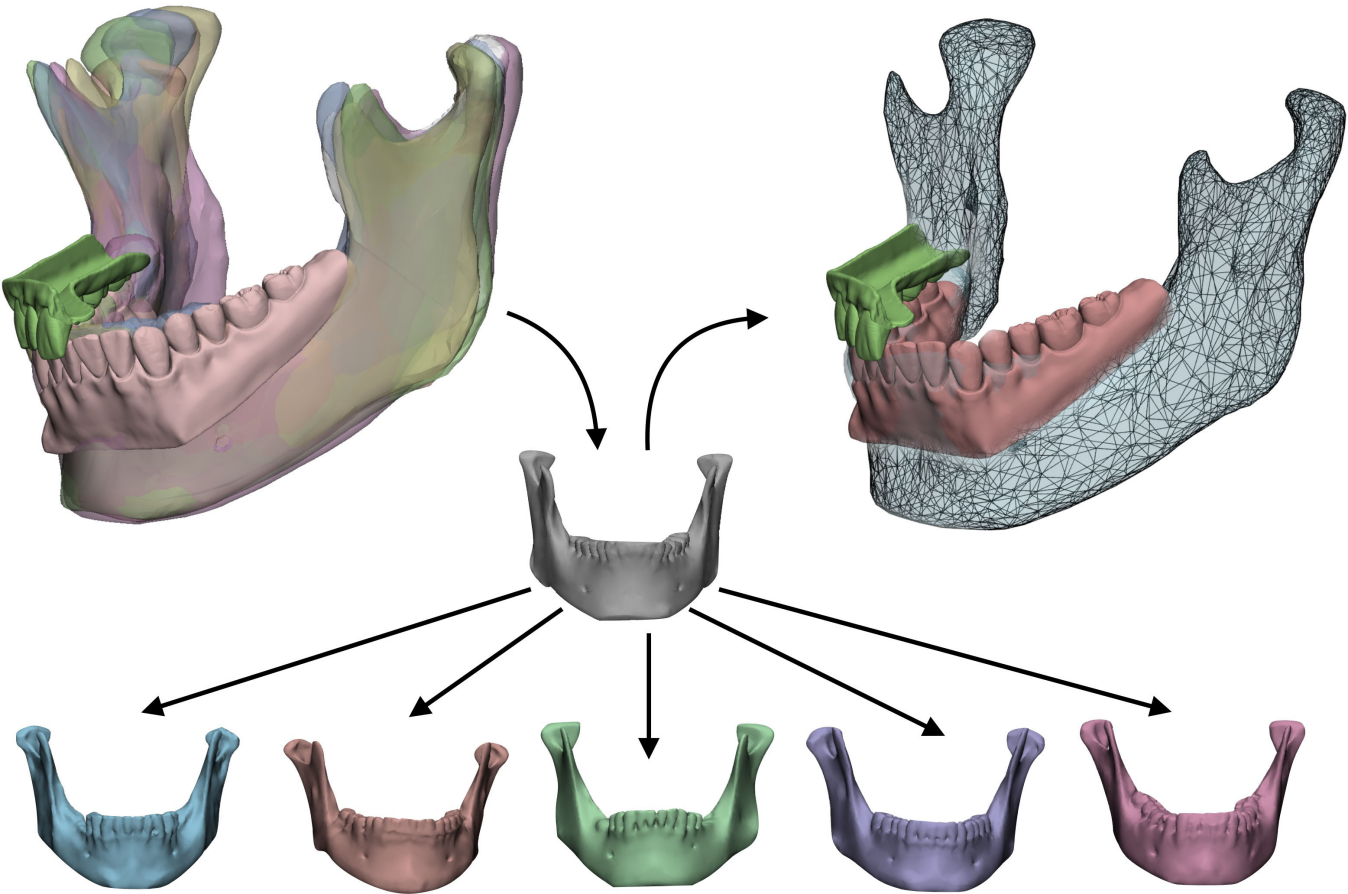
Statistical shape model (SSM) associated with digital wax-up. A target model (grey) taken from a library of STL based on anatomical resemblance with the patient is iteratively modified by applying population variance using five modificators. The result is a mesh made suitable for the actual patient which will be used to reconstruct the final shape of the mandibular prosthesis.

For cases needing a total or subtotal mandibulectomy, the Authors searched in their library of mandible STL (standard tessellation language) files the most appropriate geometry to restore the mandible and defined it as “donor”. To adapt the donor mandible to the recipient anatomy, a statistical shape model (SSM) was created by computing additional mandibles, selected on the basis of different morphologies, allowing to apply population variance on the target geometry. The resulting mesh, achieved by varying coefficients to selectively modify the SSM, was extracted as an STL file, used to define the shape of the mandible contour of the custom-made prostheses optimized for the current patient (**Figure 3**).

• Virtual wax-up and definition of the final occlusion

Intraoral scanning was aligned on the dental cusps spared from resection. Data were imported in the software DentalCAD (Exocad GmbH, Darmstadt, Germany) where a virtual occlusion was constructed. For the most complex cases undergoing a complete mandibulectomy and a half maxillectomy, mandibular arch was constructed with a complete digital approach, maximizing intercuspation of mandibular crowns with their superior antagonists. The height of crowns and the identification of an occlusal plane were essential to define the appropriate level to position the fibula free flap for mandible reconstruction.

• Pedicle choice

**Figure 4:**
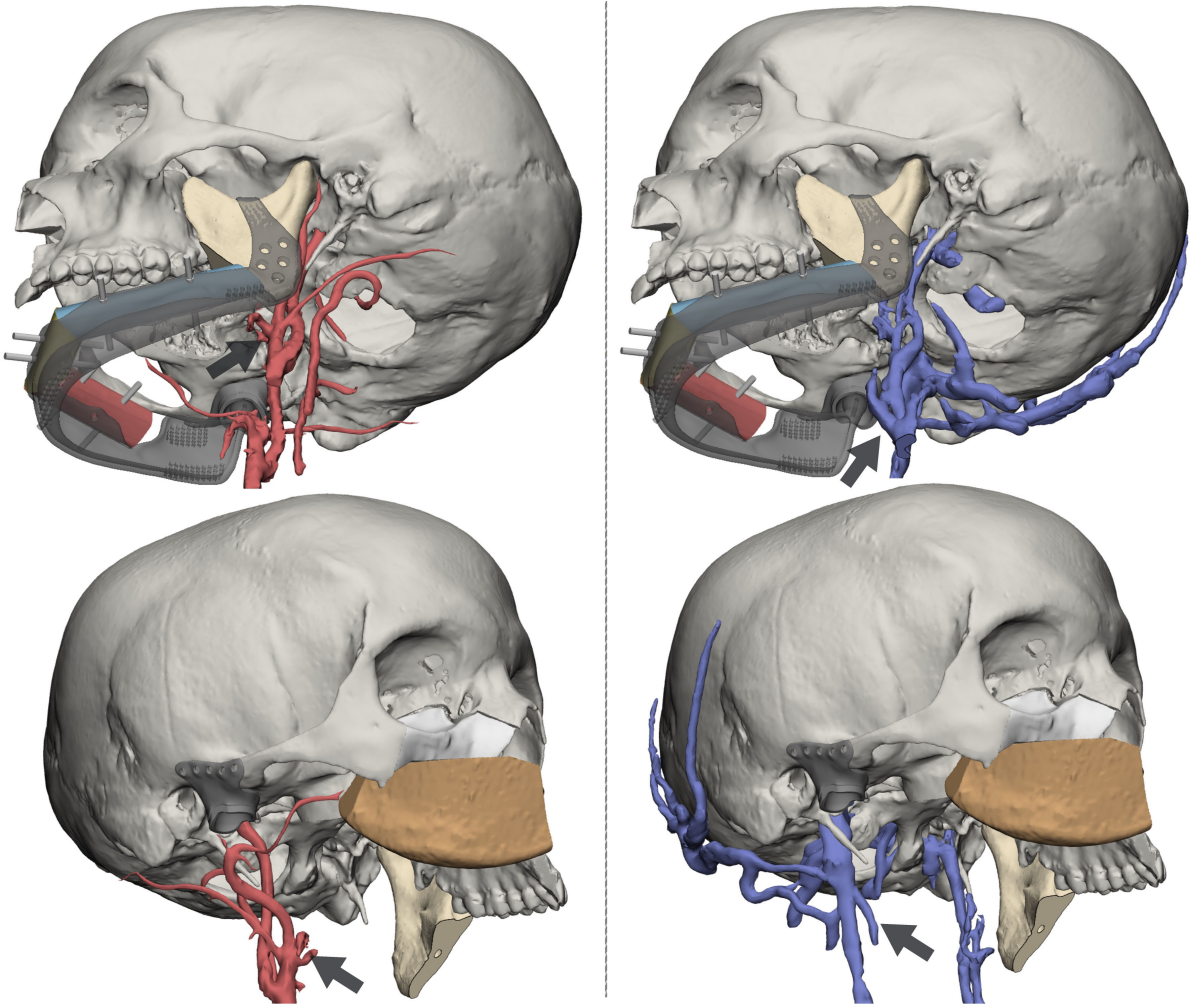
Reconstruction of arterial (left panel) and venous (right panel) vasculature from dedicated MR sequences. Black arrows identify vessels targeted for anastomosis: for the DCIA, the facial artery and the facial vein; for the fibula, the superior thyroid artery and the thyrolinguofacial venous trunk.

The 3D reconstruction of the vascular network allowed to visualize in a 3D virtual space the arterial and venous vessels. This enabled to evaluate in a patient-specific approach the relationship between the flap structure and the closest receiving vessels, enabling to identify the neck recipient trunk that requires the shortest path and the minor length of the pedicle (**Figure 4**).

• Reconstruction of the mandible (fibula surgical planning + custom implant design)

**Figure 5:**
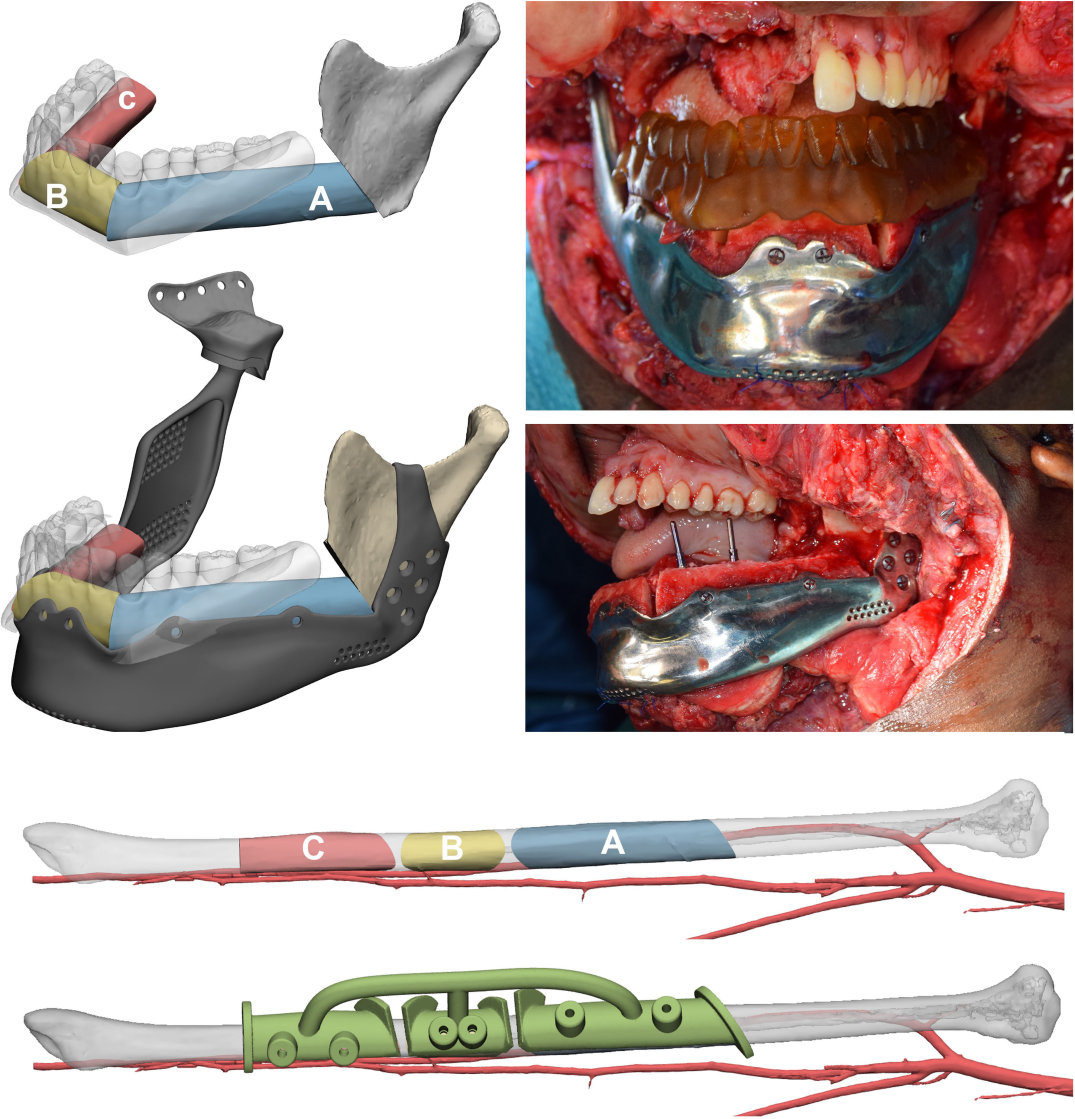
Mandibular reconstruction. The order of fibula flap segments is indicated in a proximal to distal sequence both in the mandible and in the native fibula, where a surgical guide to automate harvest was designed. Fibula segments have been positioned according to the virtual occlusion defined using the digital wax-up and their inset on a mandibular patient-specific implant designed on the SSM is simulated. Implants are placed using the 3D-printed wax-up as guide to drill holes.

The positioning of fibula flap segments was performed according to two factors: overlying dental crowns and underlying SSM mandibular border. The flap segments were positioned to be compatible with the application of implants and a future dental prosthesis, while at the same time being supported by a customized mandibular implant which included, in disarticulations, reconstruction of the temporomandibular joint (TMJ), coupled with a glenoid fossa. The fibula segments were rotated to keep the pedicle completely lingual. Once satisfied with the position of fibula segments over the mandible, they were individually aligned along the fibula axis and a surgical guide was modelled to assist the exact harvest. Segments were fixed on the customized mandibular prosthesis using the same holes provided by the guide. Microsurgical anastomoses were performed to restore blood flow and a 3D printed model of the dental wax-up was positioned to assess compatibility and define the correct implant site (**Figure 5**).

• Midface reconstruction

**Figure 6:**
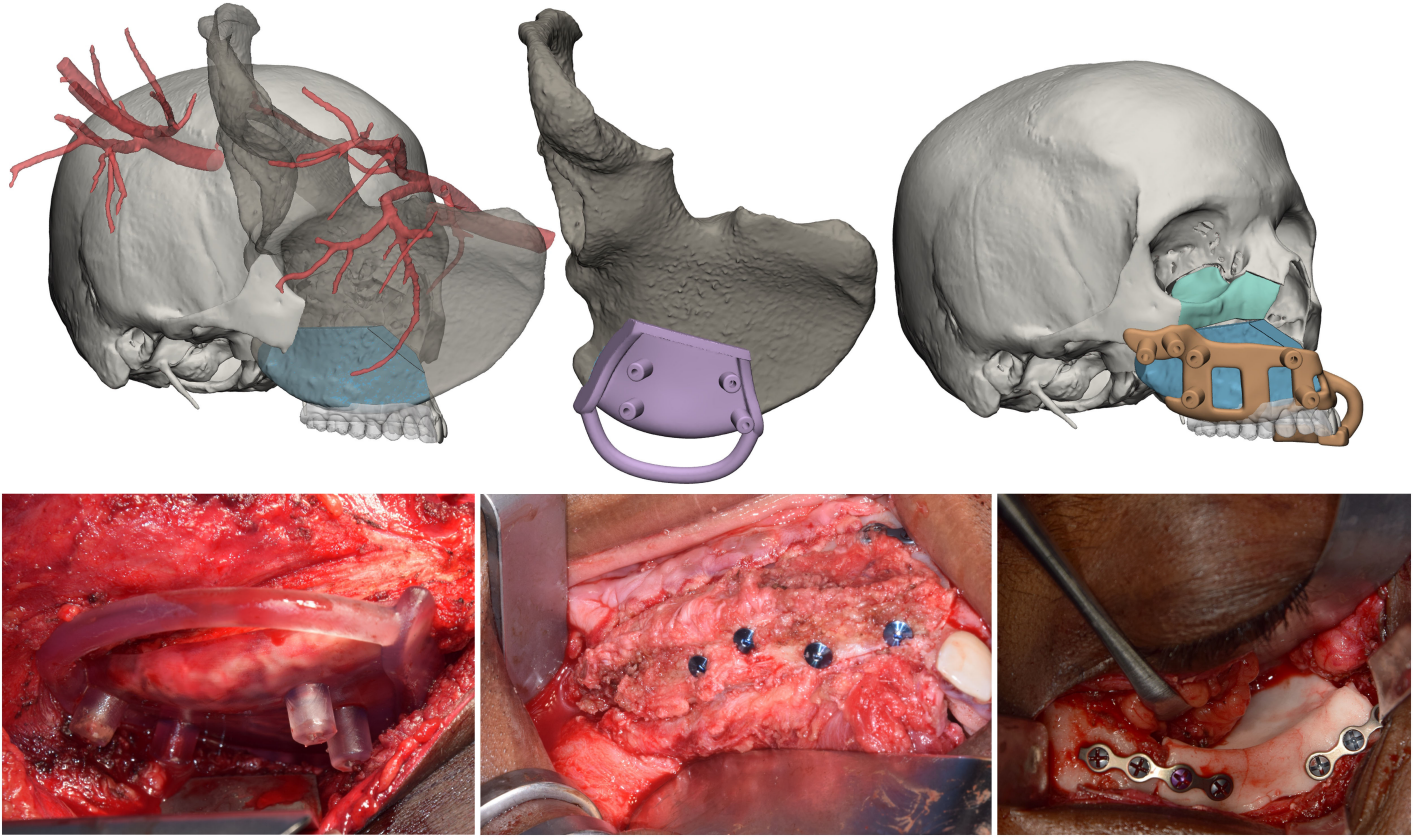
Midface reconstruction. The native iliac crest with vascularization is transposed in the defect site, where the flap is sized to ensure optimal geometrical compatibility. A surgical guide to automate the flap harvesting is designed. The flap is positioned in accordance with the digital wax-up, and a surgical guide for repositioning is designed. Finally, the orbital floor is restored by moulding a PMMA orbital implant.

The upper dental arch in occlusal relationship with its inferior antagonist was simulated in a similar way to identify a position of the midface flap compatible with implant placement and a future denture. Although also the scapular tip flap is a viable option, the deep circumflex iliac artery (DCIA) flap was chosen because it did not involve a change in patient positioning. The iliac crest was reoriented in accordance with the resection area to select the most suitable part and size. Subsequently, the simulation included further flap sculpting to trim any bone excess. Finally, an innovative surgical guide was modelled to enable precise DCIA flap insetting, holding the flap secured thanks to a custom-designed socket and at the same time allowing for fixation in the desired position. Similarly, implants were placed in the iliac crest using the 3D printed dental wax-up model (**Figure 6**).

• Orbital reconstruction

For class IV maxillectomies, the reconstruction of the orbital floor was needed. A mirroring of the contralateral integer orbit was performed to define the optimal shape of the orbital floor and ensure that the reconstruction did not alter the volume symmetry between the orbits. The implant was designed according to such principles and, using a Boolean operator, it was made correspondent with the resection edges. A molding system bearing the negative impression of the implant was designed and 3D printed for the intraoperative creation of an implantable part made of polymethyl methacrylate (6).

### 2.4 Outcome evaluation

**Figure 7:**
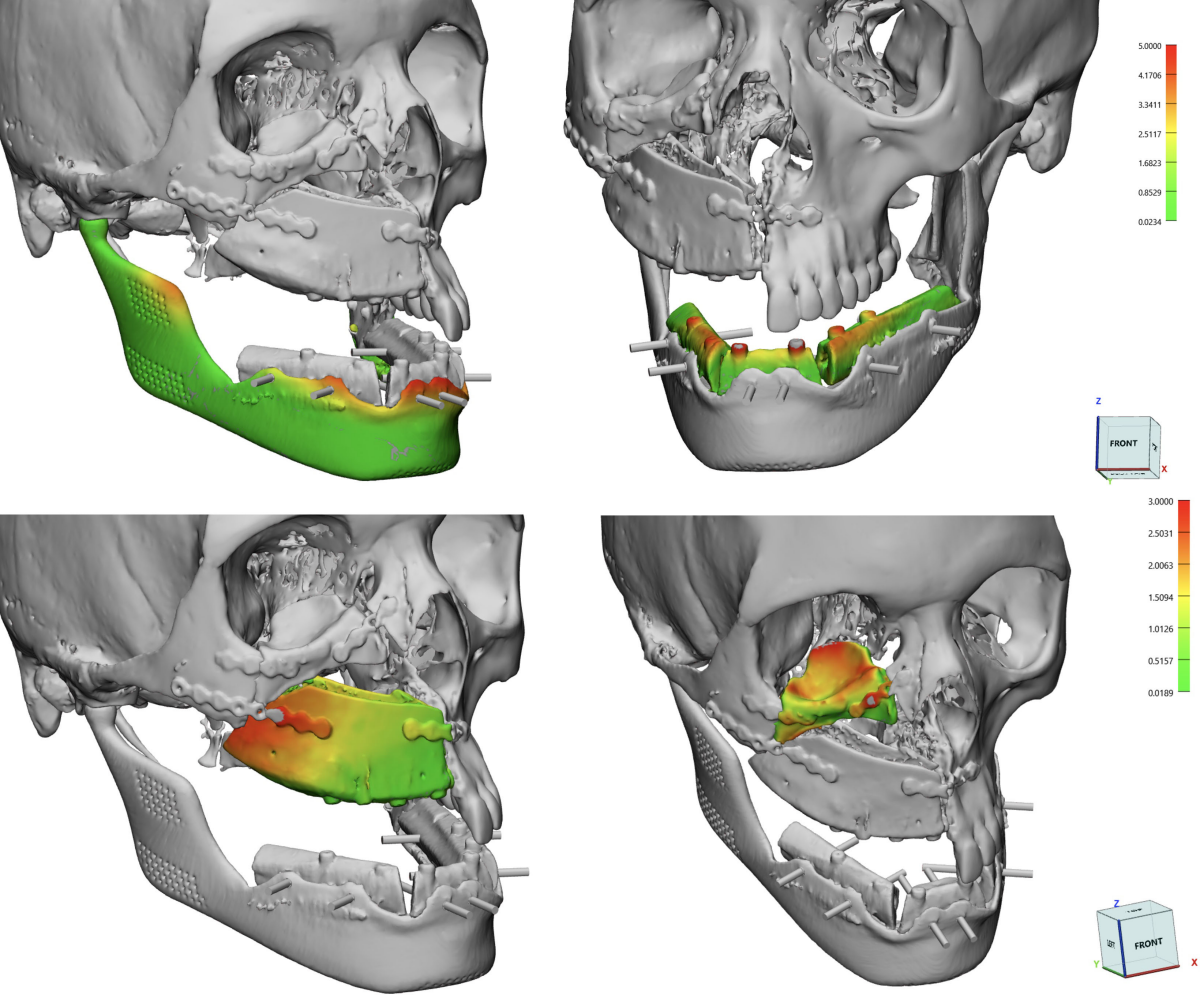
Calculations of RMSE using a surface deviation analysis for single subcomponents: DCIA and fibula flaps, mandibular implant, orbital implant.

**Figure 8:**
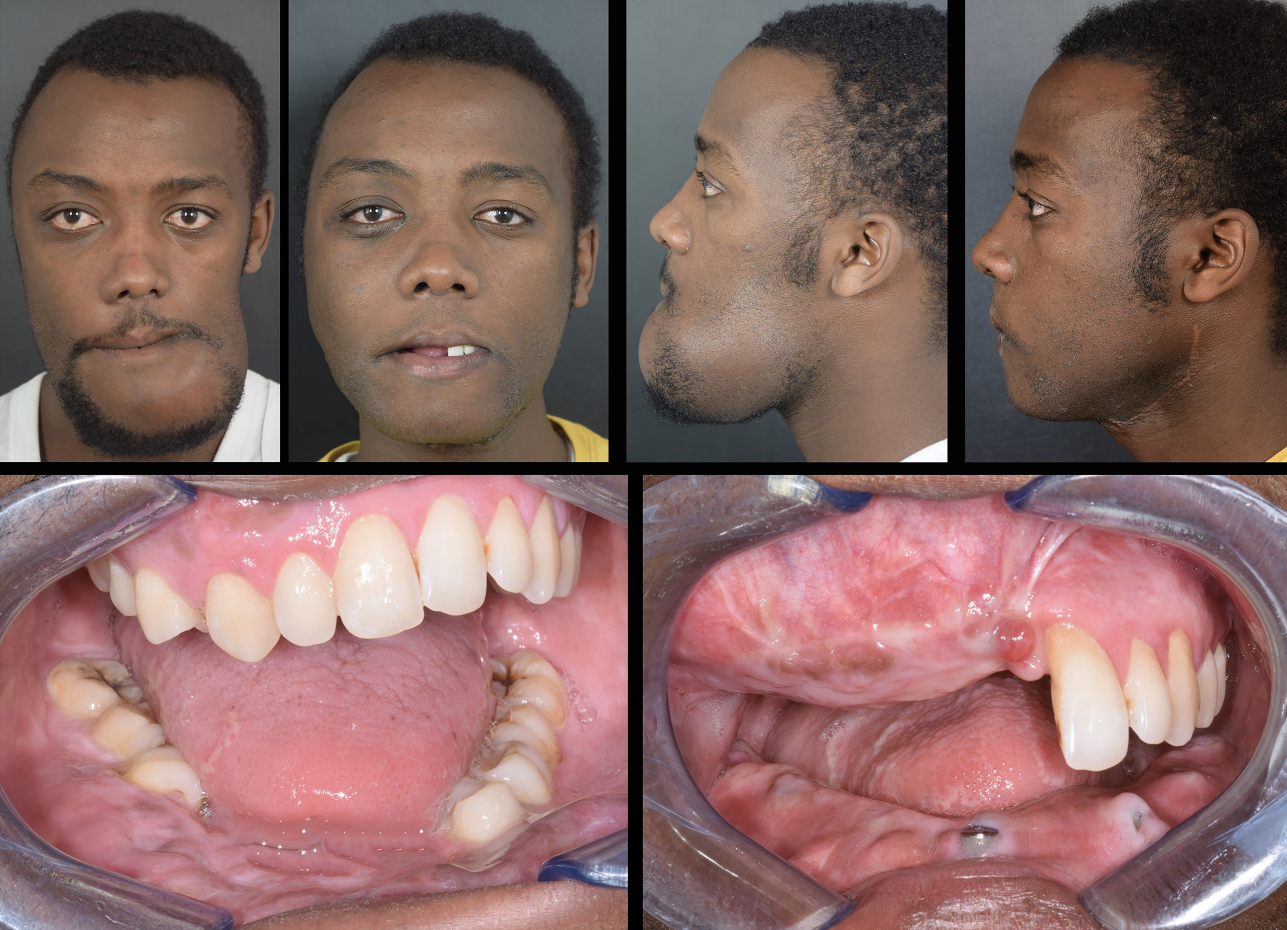
Preoperative vs 6-month postoperative appearance of the patient. Top row: full face photographs; bottom row: intraoral view showing implants placed during surgery.

Flap survival was monitored clinically every 3 hours for the first 3 days, and every 6 hours up to the first week. Two weeks after surgery, all patients underwent a postoperative CT scan with the same parameters, which was processed in Mimics to extract flaps and titanium implants from surrounding bone, yielding separate objects. Extracted objects were imported in the same coordinate system as the virtual planning project in order to estimate the deviation of the achieved surgical outcome from its planned equivalent. To quantify this, a part comparison analysis, computing Euclidean distances between pairs of aligned meshes across all their vertices was performed, and results were mapped in a color scale. The overall entity of deviation between the two geometrical entities was expressed as root mean square error (RMSE), allowing to understand the accuracy of flap and implant positioning (**Figure 7**). Clinical photographs were taken 6-month postoperatively (**Figure 8**).

## 3 Results

Clinical and demographic data of patient enrolled in this study are described in **Table 1**.

For all patients, the reconstruction of the mandible was performed using the fibula free flap, while the midface was reconstructed using the DCIA flap. There were no clinical complications concerning the harvesting sites.

The number of implants inserted in the fibula flap bone ranged between 5 and 6 for total or subtotal resection, while segmental defect, limited to less than one half of the mandible, required from 2 to 3 implants. As for the iliac crest, the implants ranged from 3 to 4 along the superior rim of the flap.

We did not perform a simultaneous dental prosthesis placement in any of the patients, as the customized mandibular prosthesis which supports the flap was not designed to withstand immediate load, nor was the iliac crest flap replacing the maxilla.

For all patients it was possible to identify the arterial trunk for the anastomosis in the virtual plan.

Duration of surgery ranged from a minimum of 13 hours to the maximum of 18 hours and involved two different teams for ablative and reconstructive surgery.

Virtual surgical planning and 3D printed guides were used in all cases according to the sequence detailed in Materials and Methods section. Average RMSE for the iliac bone crest flap was of 3.2 ± 0.36 mm; as for the fibula flap, RMSE value was of 2.3 ± 0.65 mm. As for patient-specific implants, for mandibular prostheses the average RMSE was 2.46 mm with 0.76 mm standard deviation; while there was a single orbital PMMA prosthesis in patient 1 (RMSE=2.1)

After surgery, TMJ functionality was assessed by measuring the average mouth opening, which was of 2.8 ± 0.7 mm at 3-month and 3.4 ± 0.9 mm at 6-month follow-up.

## 4 Discussion

Reconstruction of large defects involving different subunits of the facial skeleton represents one of the major challenges in cranio-maxillo-facial surgery. Multiple approaches have been proposed, and can be synthetically ascribed to the following groups:

• single chimeric bone flap
• single bone flap and single soft-tissue flap
• single bone flap and customized prosthesis

Single chimeric bone flaps offer surgeons a versatile solution to perform a composite reconstruction including multiple tissues transposed in different anatomical sites, as each flap territory is based on a vascular supply independent from the interconnection with contiguous parts, with the exception of the “mother” vessel which is anastomized to head and neck vessels. Such flap architecture allows for greater mobilization of tissue subunits, allowing to use a single flap to reconstruct composite defects: in this respect, the scapular tip flap was described a versatile technique allowing to reconstruct midfacial defects, providing at the same time a bicortical bone framework suitable for implant placement, as well as a generous muscular cuff, and eventually the possibility to harvest a myocutaneous paddle using the latissimus dorsi (7, 8). Other flaps, typically the fibula flap, are not harvested as chimeric flaps, but can be arranged in complex spatial conformations which enable the reconstruction of geometrically complex defects (9).

In the last decades, progresses in medical imaging software and additive manufacturing allowed to perform accurate virtual reality simulations to restore the missing parts of the craniofacial skeleton, using digital techniques such as mesh mirroring, polysculpting and CAD-design. The concomitant advancement in metal 3D printing, including technologies such as selective laser sintering (SLS), melting (SLM) and electron-beam melting (EBM) made possible to manufacture complex craniofacial implants that accurately reproduce the native anatomy. Notably, these techniques have been variably combined with microsurgical approaches, leading to the assembly of microsurgical flaps over customized implants, to provide the most trustful restoration of the skeletal framework. Such approaches have been implemented in particular for orbito-maxillary resections, providing an effective method to restore the anatomical integrity of the orbit, while at the same time supplying a soft tissue lining that keeps the implant covered and eventually a native bone component to insert dental implants (10).

Although chimeric flaps represented a significant improvement in microsurgery thanks to the possibility to use a single vascular supply to provide tissue to different anatomical areas, they have a limited indication for very large defects that simultaneously involve the middle and the inferior third of the maxillofacial skeleton. According to Mannelli and colleagues (11), intrinsic chimeric flaps are contraindicated for large soft tissue need (>350 cm^2^) and large and complex bone defects (>13-14 cm). Moreover, chimeric flaps require a higher microsurgical training compared with simple flap harvesting, and require the ablative surgical time to be complete prior to harvesting the flap, thus precluding any dual-team surgery, with an increase in surgical time (12). In these cases, several papers have explored the possibility to perform a double microsurgical procedure, which involves the simultaneous harvest and inset of two different microsurgical flaps. Some Authors have collected literature evidence on the use of dual free flap in head and neck reconstruction, concluding that combinations of flaps generally involve a bone and a soft tissue flap, or two soft tissue flaps, in particular, the predominant combination for mandible reconstruction involves the simultaneous use of the fibula free flap with the anterolateral thigh flap (1, 13). However, the simultaneous use of two osseous free flaps is seldom reported (14), with no previous reports considering the combination of the DCIA and the fibula flap to restore a midfacial and mandibular defect.

In this paper, we report our preliminary experience with a dual osseous flap reconstruction of broad midfacial and mandibular defects using the combination of DCIA and fibula flap, presenting an entirely digital workflow which has several benefits, including: sequencing and strategy; surgical automation, functional restoration, accuracy. First, the precise definition of preoperative anatomy plays a crucial role, and it is of prominent importance to instruct radiologists to acquire images with well-defined protocols for 3D reconstruction to maximize the ease and accuracy of segmentation. In particular, as reported by the same Authors, the use of dedicated MR sequences with maximal spatial resolution parameters allows to perform a differential segmentation which can discriminate between arterial and venous blood flow, leading to trustful models of vasculature that facilitate the visualization of recipient head and neck vessels (5). Similar reconstructions are performed also for the donor region of flaps, enabling to define the length of their pedicle to anticipately establish the most appropriate vascular connection. Another innovative approach is the definition of the ideal shape of the mandible in cases where the preoperative deformation conceals any intelligible shape corresponding to the premorbid condition. This was achieved using a statistical shape model (SSM) applied to a closest-donor template mandible chosen from a library based on similar antropometric features (15) combined with organic mesh modeling to adapt the SSM into optimal articulation with the glenoid fossa. SSM was used as source shape to design the final mandible implant to restore the most accurate shape of the mandibular border, while at the same time supporting the fibula free flap accommodated within a custom-designed socket (16). Likewise, the arrangement of fibula segments was not only guided by the SSM, but also by occlusion: it is important to adopt an occlusion-driven approach to flap positioning, taking into account the final position of dental crowns in their optimal intercuspation with superior antagonists and modifying the height of flap inset according to the desired occlusal plane (17–19). For this purpose, the possibility to recreate all missing dental crowns within a virtual wax-up model was essential to define occlusal relationship and posing, together with the SSM, an additional constraint for optimal flap positioning. As mentioned, given the complexity of such surgeries, simultaneous prosthesis placement with immediate load was not performed in such cases, although it was described for single flap surgeries (17), as it might compromise the stability of flaps. We preferred to delay dental prosthesis placement at 8-12 months after surgery to ensure maximal osteointegration and stability.

For mandibulectomies with condyle disarticulation, TMJ reconstruction was included in the final implant: although for some Authors the condyle is sufficient to restore the function of TMJ (20), we recommend coupling the condyle with a prosthetic glenoid fossa to yield a fully functional TMJ. The same principles were applied to DCIA flap, which was positioned according to an optimal relationship with occlusion. The choice of the iliac crest free flap was based on the possibility of harvesting the flap without a position change for the patient, allowing multiple surgical teams to simultaneously work; moreover, the iliac crest provides a wide, thick-cortical surface for stable implant placement. Virtual surgical planning of iliac crest positioning enabled to perform a detailed osteometric study to establish the most favorable insetting position for implant placement (21). At the same time, a customized orbital implant overlying the DCIA flap was intraoperatively created by PMMA moulding

The fully digital workflow implemented by this study enabled to define a detailed surgical sequence and was performed entirely by the surgical team; likewise, any surgical guide was 3D printed in-house. The advantages of complete clinical management of the virtual planning involve the awareness of surgical accesses, thorough study of the clinical case and virtual interaction with the digital model. The surgical automation allowed by the entirely digital workflow allowed to decrease surgical time for flap shaping and insetting, facilitating all maneuvers related to the geometrical reconstruction of the maxillofacial skeleton using an entirely in-house workflow, with surgical guides for resection, fibula flap and DCIA flap harvest, DCIA flap positioning, as well as a customized mandibular implant shaped on the fibula flap and the SSM (22). In terms of surgical accuracy, the automation of surgical sequence enabled to restrict positional error for flaps and implants within 4 mm for maxillary reconstruction and 2 mm for the neo-mandible, representing a substantial correspondence between the planning and surgical outcome, thus meeting the requirements for functional restoration.

## 5 Conclusion

This paper reports a preliminary experience in facial reconstructive surgery using the simultaneous combination of two osseous flap in an entirely computer-guided sequence with meticulous study of osteometric and vascular features of flaps and their recipient sites. Virtual planning allowed to match needs in reconstructive properties with functional demands, allowing to tailor flap design and inset on the requirements for an effective occlusion, including temporomandibular joint replacement as well. Virtual surgical planning is an essential part of each reconstructive strategy in a contemporary vision of maxillofacial surgery, especially for complex cases, allowing to precisely define a great number of preoperative variables, to design and manufacture guides that assist the surgeons in accuracy-demanding procedures. Most importantly, the whole sequence of planning was designed by surgeons, that mentalized and reproduced it in the operating room. In conclusion, double bone free flap is a valuable resource to reconstruct wide defects that simultaneously involve two thirds of the cranio-maxillo-facial skeleton, but an extended virtual planning study should be always performed before approaching this surgical option.

## Data availability statement

The raw data supporting the conclusions of this article will be made available by the authors, without undue reservation.

## Ethics statement

The studies involving human participants were reviewed and approved by IRB_45_2020. The patients/participants provided their written informed consent to participate in this study. Written informed consent was obtained from the individual(s) for the publication of any potentially identifiable images or data included in this article.

## Author contributions

AT designed the study, performed virtual surgical planning, created virtual models, 3D printed models, and wrote the full paper. DB acquired radiologic images with required protocols and wrote the imaging part of this paper. SF read and approved the manuscript. MR coordinated the research team and approved the final manuscript before submission. All authors contributed to the article and approved the submitted version.
